# Investments

**Published:** 1872-04

**Authors:** 


### INVESTMENTS.

MANY a heart pang would be prevented if every man and woman in civilized society were able to lay aside money enough before fifty, the interest of which would yield quarterly twenty-five dollars. Go to the Presbyterian Home for old women, at the Central Park, built by the wise munificence of James Lenox, and see widows who once lived in wealth and luxury, now fed and sheltered by the generosity of others.

A loving son wrote the other day, " I found mother quite comfortable, for her. She was eighty-six yesterday." He had spent considerable sums for many years past for medical aid and otherwise, and was still working hard, and with many self-denials was endeavoring, in all possible ways, to have life's close a day of sunshine to her. We could not help writing to him of such an investment, " Better than laying up in any savings-bank on earth, is the money you expend for the comfort of your mother's old age; it is treasure deposited in the Bank of Heaven, to bear prompt and large-paying interest forever! The more so, as the money thus laid out by you is earned from time to time in laborious and anxious industries." Sweet, beyond the food of angels, and like a perennial spring of health, to life's latest hour, are the thoughts of sacrifices made for " Father " and " Mother " before they passed from the stage of life; and yet they can never be repaid for the loving care shown us through all the long years of infancy, childhood, and youth.

